# Total and cause-specific mortality associated with meat intake in a large cohort study in Korea

**DOI:** 10.3389/fnut.2023.1138102

**Published:** 2023-03-13

**Authors:** Anthony Kityo, Sang-Ah Lee, Daehee Kang

**Affiliations:** ^1^Department of Preventive Medicine, Kangwon National University School of Medicine, Chuncheon, Republic of Korea; ^2^Interdisciplinary Graduate Program in Medical Bigdata Convergence, Kangwon National University, Chuncheon, Republic of Korea; ^3^Department of Preventive Medicine, Seoul National University College of Medicine, Seoul, Republic of Korea

**Keywords:** meat intake, processed red meat, all-cause mortality, cancer mortality, CVD mortality, cohort study, cox model

## Abstract

**Background:**

Asia has experienced a large increase in meat intake in the past decade, yet the health impact of meat intake is not well studied.

**Objective:**

We examined the association of meat intake with all-cause, cancer and cardiovascular disease (CVD) mortality in an Asian country.

**Methods:**

Participants were 113,568 adults with dietary data at recruitment (2004–2013) of the Health Examinees-Gem (HEXA-G) study, a prospective cohort study conducted in 8 regions of Korea. Participants were followed until 31 December 2020. Total, red, white, and organ meat intake were computed based on a 106-item questionnaire. Multivariable Cox proportional hazard models were implemented using the lowest quintile of meat intake as the reference category.

**Findings:**

For 1,205,236 person-years, 3,454 deaths were recorded. High intake of processed red meat was positively associated with all-cause mortality [men: hazard ratio (HR) 1.21, 95% confidence interval (95% CI) 1.07–1.37; women: HR 1.32, 95% CI 1.12–1.56]. Increased risk of all-cause mortality (HR 1.21, 95% CI 1.05–1.39) and cancer mortality (HR 1.24, 95% CI 1.03–1.50) was observed in women with high intake of organ meat. Moderate intake of pork belly was associated with reduced risk of all-cause mortality in men (HR 0.76, 95% CI 0.62–0.93) and women (HR 0.83, 95% 0.69–0.98) but high intake was associated with increased risk of CVD mortality in women (HR 1.84, 95% CI 1.20–2.82). Low beef intake decreased the risk of CVD mortality in men (HR 0.58, 95% CI 0.40–0.84), but roasted pork increased cancer mortality in women (HR 1.26, 95% CI 1.05–1.52).

**Conclusion:**

There was increased risk of all-cause mortality associated with intake of processed red meat in men and women, increased risk of all-cause and cancer mortality with intake of organ meat in women, and increased risk of cancer mortality with intake of roasted pork intake in women. High intake of pork belly increased the risk of CVD mortality in women, but moderate intake was inversely associated with mortality from all-causes in both men and women.

## 1. Introduction

Estimates from the 2022 Global Nutrition Report indicated that close to two-thirds of avoidable deaths were attributed to sub-optimal dietary composition, including 9% attributed to high intake of meat, and 8% attributed to processed meat (1). The Asian region has experienced the third largest increase in red and processed red meat intake in the past decade (1), suggesting that this region is experiencing a nutritional transition toward a Westernized dietary pattern. Nonetheless, various aspects of the traditional dietary pattern that emphasize high intake of plant foods and moderate intake of fat have been retained in Asian countries like Korea (2, 3). Accordingly, there is need to understand the health impacts of meat intake in Asian countries considering their unique dietary culture. Cohort studies and meta-analyses have reported a positive association of red and processed meat intake with all-cause and cause-specific mortality mainly in Europe and North America (4–12).

However, pooled data from Asian countries indicated an inverse association between red meat intake and mortality (13). Conversely a recent cohort study from Japan reported an increased risk of total and CVD mortality with heavy intake of total and red meat, a low risk of stroke mortality with high intake of total meat and a reduced risk of all-cause and cancer mortality in men with moderate intake of processed meat (14). Different results between Asian and Western countries have been explained by low intake of meat in Asian vs. Western countries (11, 13, 14). However, red and processed meat intake in an Adventist population with low intake of meat, increased the risk of mortality (4). Previous studies in Asian populations neither differentiated processed and unprocessed red meat (13), nor accounted for methods of preparation which are also different from those in the West (15–17). On the other hand, the association between white meat consumption and mortality is inconclusive with some studies reporting inverse associations (7, 8, 10, 13, 14) and others reporting null associations (9, 11, 12).

The Health Examinees (HEXA) cohort is a large prospective study of over 170,000 adults aged above 40 years who were recruited from 38 regions of Korea between 2004 and 2013. Using this data set, we comprehensively examined meat intake by type, degree of processing, and method of preparation in relation to death from all-causes and specific causes.

## 2. Materials and methods

### 2.1. The Health Examinees-Gem Study

We conducted an observational analysis of the HEXA cohort, a prospective population-based sub-cohort within the Korean Genome and Epidemiology study (KoGEs) that was established to investigate the etiological factors of complex diseases (18). The HEXA recruited participants between 2004 and 2013 at 38 health examination centers and training hospitals located in the eight regions of Korea. The study details have been published elsewhere (19). Ethical approval was obtained from the Ethics Committee of the Korean Health and Genomic Study of the Korean National Institute of Health and the Institutional Review Boards of all participating hospitals (IRB no. E-1503-103- 657). All participants provided written informed consent before their participation.

### 2.2. Measures

#### 2.2.1. Assessment of dietary intake

Dietary intake was assessed once at baseline using a 106-food item semiquantitative food frequency questionnaire (SQFFQ) that had been tested for reproducibility and validity using 12-day dietary records obtained from 124 participants (18). Food consumption frequencies were classified into nine levels (from “never” to “three times or more a day”), and portion sizes were classified into three levels (one-half, one, and one and a half servings). Energy and macronutrient content of each item was estimated using a food composition table developed by the Korean Rural Development Administration (RDA) (19). Meat items on the SQFFQ were pork (roasted and braised), and pork belly; edible viscera/organ meat; processed meat; beef (steak/roasted), beef soup with bones, and beef soup with vegetables; dog meat; and chicken (fried/stew). For mixed dishes which contained meat, we extracted meat weight by applying weights using the food recipe information. The applied weights represent the % weight contributed by a meat item to the mixed dish. Red meat included beef, pork, and dog meat while white meat included chicken.

#### 2.2.2. Baseline covariates

Educational level, and household income were assessed in addition to demographic characteristics such as age and sex. Other covariates included lifestyle factors such as: smoking, where current smokers were defined as participants who had smoked more than four hundred cigarettes during their lifetime and were still smoking (20); drinking-categorized into current alcohol drinkers, past drinkers and never drinkers. Current alcohol drinkers were those who reported that they had ever drunk alcohol and were still drinking at the time of the interview. Regular physical exercise was assessed by asking participants to report (1) whether they engage in regular physical exercise that causes body sweating; (2) the number of times they engage in these exercises in a week (1–2 times/week to everyday); and (3) the duration of the exercise. Regular exercise was defined as engaging in activities that caused body sweating for at least five times a week lasting at least 30 min per session.

Weight and height were objectively measured at baseline by trained medical staff. Body mass index (BMI) was calculated as weight in kilograms divided by the square of height in meters (kg/m^2^). BMI was categorized into four classes based on the WHO classification of BMI for Asian adults: < 18.5, 18.5–22.9, 23–24.9, 25.0–29.9, and ≥30.0 kg/m^2^ (21). The information about diseases and use of medication was reported by participants through a standardized questionnaire that was administered by trained staff, and was used to define prevalent cancer, cardiovascular, cerebral vascular, respiratory and gastrointestinal diseases. Chronic kidney disease was diagnosed using estimated glomerular filtration rate (eGFR < 60 mL/min/1.73 m^2^) that was estimated using the Chronic Kidney Disease Epidemiology Collaboration equation (CKD-EPI) (22). Diabetes was defined as fasting blood glucose ≥126 mg/dL or drug treatment for elevated fasting blood glucose, hypertension was defined as systolic blood pressure ≥130 mmHg or diastolic blood pressure ≥85 mmHg or drug treatment for elevated blood pressure. Abdominal obesity was defined as waist circumference ≥90 cm for men and ≥80 cm for women (23). The National Cholesterol Education Program Adult Treatment Panel III (NCEP-ATP III) criteria was used to define metabolic syndrome (24). For each chronic disease, participants were assigned a score of 1 (one) in the presence and a score of zero (0) in the absence of each disease. These scores were summed across all the diseases to create a disease score. The disease score was then classified as: zero (no disease) and ≥1 (having atleast a disease).

#### 2.2.3. Ascertainment of mortality

The date and causes of death from 2004 to 31 December 2020, were ascertained through linkage to the death certificate data base of the Korean National Statistical Office. The deaths of participants on Medicaid were ascertained through linkage to the National Health Insurance Service. Participants' unique identifiers were used to add mortality data from Statistics Korea. For cause-specific mortality, the 10^th^ revision of the International Classification of Disease (ICD-10) codes was used. ICD-10 code C00-C97 and I00-I99 were used to classify cancer and CVD-specific deaths, respectively.

#### 2.2.4. Statistical analysis

Participants were divided into sex-specific quintiles of total meat intake and intake of meat subtypes. The main outcome variable was death from any cause, cancer or CVD. Missing data were replaced by the mode for categorical variables and by the median for BMI. The distribution of participant characteristics according to quintiles of total meat intake was described using percentages for categorical variables or least square means for continuous variables and was stratified by sex.

Follow-up time was calculated for each participant starting from the date of recruitment until the date of death. Participants who did not experience the event were censored on 31^st^ December 2020. We examined the association of meat intake with all-cause, cancer and CVD mortality using multivariable cox proportional hazard models. Adjusted hazard ratios (HRs) and 95% confidence intervals for meat intake, using quartile one (Q1) as the reference category, were computed and adjusted for age (continuous), demographic factors (marital status, educational level, job, and monthly household income); lifestyle factors (total energy intake, smoking, drinking, and regular physical exercise); and health-related factors (BMI and history of chronic disease). The proportional hazards assumption was assessed by including multiplicative terms between variables and follow-up time and was not violated (*p* > 0.05 for all categories).

We stratified the analyses by sex since meat intake is higher in men than women. In addition, we conducted sensitivity analysis (1) by excluding participants who died within 1 year of follow-up, to avoid latent period bias and reverse causation; (2) adjusting for alcohol intake in grams/day (continuous) to test residual confounding from alcohol intake; and (3) adjusting for selected dietary variables that are associated with meat intake (vegetables, fish, seafood, and legumes).

All data were analyzed using SAS software version 9.4 (SAS Institute Inc., Cary, NC, USA), and P < 0.05 was used to define statistical significance.

## 3. Results

**Figure 1:**
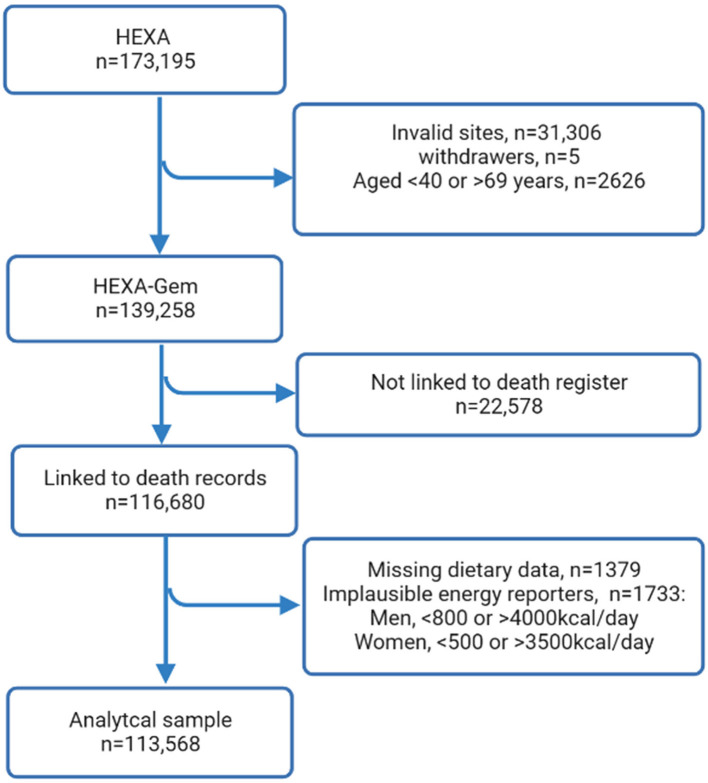
Selection of study participants.

The HEXA-Gem (HEXA-G) (*n* = 141,968) sample was derived from the original HEXA study (*n* = 173,195) by excluding: (1) sites that only participated in the pilot study from 2004 to 2006; (2) sites that did not meet the HEXA standards for biospecimen quality control; (3) sites that participated in the study for < 2 years (25) (*n* = 31,306), those who withdrew from the study (*n* = 5), and those younger than 40 or older than 69 years (*n* = 2,626). A total of 22,578 participants did not consent to record linkage and were excluded from the analysis. Furthermore, 3,112 participants were excluded due to missing data on dietary intake (*n* = 1,379) and implausible energy reporting (*n* = 1,733) leaving 113,568 participants for primary analysis (Figure 1).

The mean age at baseline was 53.5 years (SE 8.2), 38,847 (34%) were men and 74,721 (66%) were women. For 1,205,236 person-years [median follow-up of 10.6 (9.5–11.9) years], 3,454, 1,720, and 539 total, cancer and CVD deaths, respectively, were recorded. The highest consumers of total meat were more likely to be younger, highly educated, and had high income. In addition, individuals in the highest quartiles of total meat intake were current drinkers, less likely to engage in regular exercise and had a higher BMI in men and low BMI in women (Table 1).

**Table 1:** General characteristics of participants according to quintiles of total meat intake in the HEXA-G study.

	**Quintiles of total meat intake (g/day)**								
	**Men**					**Women**				
	**Q1**	**Q2**	**Q3**	**Q4**	**Q5**	**Q1**	**Q2**	**Q3**	**Q4**	**Q5**
*n*	7,756	7,783	7,769	7,770	7,769	14,945	14,943	14,942	14,940	14,951
Age, years, mean ± SEM	56.8 ± 0.1	54.6 ± 0.1	53.2 ± 0.1	52.3 ± 0.1	51.7 ± 0.1	55.4 ± 0.1	53.4 ± 0.1	52 ± 0.1	51 ± 0.1	50.3 ± 0.1
Education, ≥college	35.4	40.3	43.9	45.4	44.2	16.3	20.0	23.5	27.0	27.8
Income, ≥3,000 USD	37.6	44.5	49.5	51.0	51.3	29.2	36.6	42.0	46.3	46.2
Married	94.1	94.3	95.0	94.0	94.3	82.3	86.4	88.0	89.1	90.5
Seoul resident	32.8	37.9	39.5	38.8	36.3	30.2	33.7	35.7	37.3	34.5
Current smoker	44.0	42.6	41.4	40.1	37.7	1.2	1.1	1.2	1.3	1.4
Past smoker	24.4	28.8	31.7	33.8	37.8	1.9	1.9	2.4	2.3	2.9
Current drinker	60.1	70.7	76.4	77.0	79.6	19.6	27.6	32.3	35.8	38.6
Past drinker	10.9	7.8	6.0	5.4	5.4	2.2	1.8	1.7	1.8	1.8
Regular exercise	39.4	36.0	36.0	34.4	33.5	38.1	37.1	36.1	35.2	33.1
BMI, ≥25.0 kg/m^2^	36.7	37.8	39.4	41.3	43.9	30.1	28.5	27.6	27.1	26.9
**History of disease**										
Cancer	3.6	2.4	1.9	1.7	1.6	5.6	4.4	3.2	3.0	2.7
Hypertension	54.5	53.2	51.8	52	50.9	44.5	38.9	36.1	33.3	32.2
Diabetes	12.9	11.7	10.5	9.6	10.7	7.9	5.8	5.4	4.7	4.6
Metabolic syndrome	20.4	19.8	20.0	20.2	20.5	23.6	19.5	17.9	16.0	15.6
CKD	7.5	6.5	5.6	5.6	5.3	7.5	6.5	5.6	5.6	5.3
Heart disease	1.5	0.9	0.7	0.8	0.6	0.8	0.4	0.3	0.3	0.2
Disease score, 1	6.8	5.0	4.1	3.8	3.0	6.8	5.0	4.1	3.8	3.0
Total deaths, *n*	566	424	372	396	308	395	278	264	228	223
Cancer deaths, *n*	251	199	176	191	135	198	152	151	137	130
CVD deaths, *n*	86	63	65	69	50	62	40	36	35	33
Energy intake, Kcal/d, mean	1569.8	1682.1	1801.6	1941.8	2214.9	1423.5	1,528	1634.5	1766.8	2044.3
Total meat, g/d, median	12.7	28.2	44.3	68.7	123.1	6.3	17.5	29.8	48.8	94.3
Menopausal women						77.3	68.0	60.6	55.4	51.5
Hormonal therapy user						4.5	4.2	3.9	4.0	3.7
Oral contraceptive user						82.0	82.5	83.5	84	84.6

Values are % unless otherwise indicated. CKD, chronic kidney disease.

Total energy intake was high in the highest quintile of meat intake. The proportion of individuals with chronic diseases, menopausal women, and users of postmenopausal hormonal therapy was low among highest consumers of total meat (Table 1). In addition, vegetable, fish, and sea food intake were high among highest consumers of total meat (Supplementary Table 1). The distribution of meat intake by sex are displayed in Supplementary Table 2. The median intake of total meat was 44.3 g/day in men and 29.7 g/day in women. Red meat contributed over 90% to total meat intake, and 98% of red meat intake was unprocessed in both genders.

**Figure 2:**
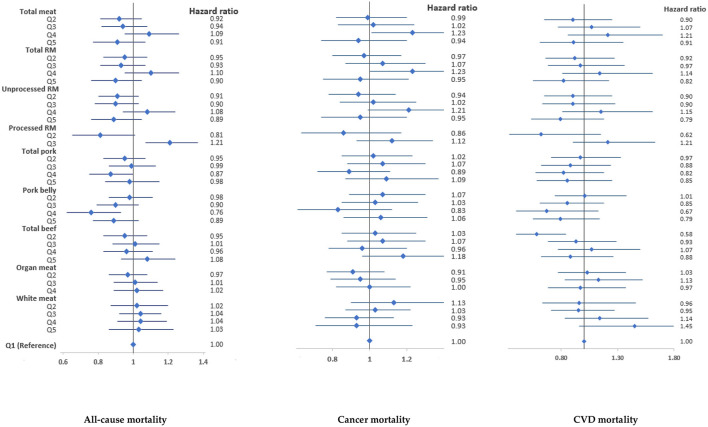
Associations of meat intake with all-cause and cause-specific mortality in the HEXA-G study in men. Points are hazard ratios, and lines represent 95% confidence interval. Models were adjusted for baseline age, marital status, education, income, job, smoking, drinking, regular physical exercise, total energy intake, total meat intake (for specific meat types), history of chronic diseases, and body mass index. RM, Red meat. 95% confidence intervals, sample sizes and person years are shown in Supplementary Table 3.

**Figure 3:**
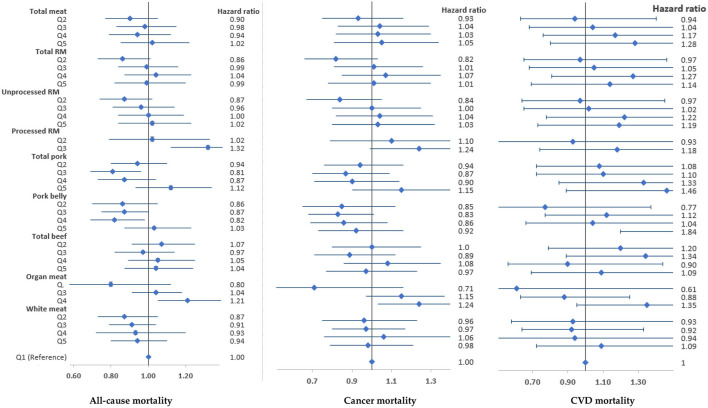
Associations of meat intake with all-cause and cause-specific mortality in the HEXA-G study in women. Points are hazard ratios, and lines represent 95% confidence interval. Models were adjusted for baseline age, marital status, education, income, job, smoking, drinking, regular physical exercise, total energy intake, total meat intake (for specific meat types), history of chronic diseases, body mass index, and menopausal status. RM, Red meat. 95% confidence intervals, sample sizes and person-years are shown in Supplementary Table 4.

Total meat, total red meat or white meat intake was not associated with all-cause mortality in both men and women (Figures 2, 3). However, high intake of processed red meat was positively associated with all-cause mortality in men [hazard ratio (HR) 1.21, 95% confidence interval (95% CI) 1.07–1.37] and women (HR 1.32, 95% CI 1.12–1.56). Moderate intake of pork belly was inversely associated with all-cause mortality (men, HR 0.76, 95% CI 0.62–0.93; women, HR 0.82, 95% CI 0.69–0.89) (Figures 2, 3), but high intake was positively associated with CVD mortality in women (HR 1.84, 95% CI 1.20–2.82) (Figure 3). There was a low risk of CVD mortality in men with low beef intake (HR 0.58, 95% CI 0.40–0.84). However, women with high intake of organ meat/meat viscera had a high risk of all-cause (HR 1.21, 95% CI 1.05–1.39) and cancer mortality (HR 1.24, 95% CI) 1.03–1.50 (Figure 3). These associations persisted in sensitivity analyses (Supplementary Tables 7, 8).

When the analyses were extended to meat intake according to preparation method, a high risk of cancer mortality was observed in women with the highest intake of roasted pork (HR 1.26) (Table 2).

**Table 2:** Meat intake, all-cause mortality and cause-specific mortality according to meat preparation methods in the HEXA-G study in men.

	**All-cause mortality**			**Cancer mortality**		**CVD mortality**	
**Men**	**Person-years**	**Deaths**	**HR (95% CI)**	**Deaths**	**HR (95% CI)**	**Deaths**	**HR (95% CI)**
Roasted pork	89,519	362	0.95 (0.82–1.09)	163	0.92 (0.75–1.13)	57	0.81 (0.57–1.14)
Braised pork	100,294	416	1.06 (0.94–1.19)	197	1.12 (0.94–1.33)	69	1.05 (0.78–1.42)
Beef steak	106,160	448	0.97 (0.86–1.10)	221	1.06 (0.89–1.27)	66	0.83 (0.60–1.13)
Beef soup	126,615	483	1.00 (0.89–1.13)	225	0.93 (0.78–1.11)	85	1.01 (0.76–1.36)
Beef in vegetable soup	85,649	404	1.01 (0.88–1.15)	136	0.99 (0.81–1.20)	69	1.12 (0.80–1.57)
Fried chicken	131,345	505	1.02 (0.90–1.17)	163	0.96 (0.79–1.17)	94	1.25 (0.91–1.72)
**Women**							
Roasted pork	240,774	362	1.10 (0.95–1.26)	222	1.26 (1.05–1.52)	53	1.16 (0.81–1.67)
Braised pork	327,391	191	1.05 (0.93–1.19)	284	1.08 (0.92–1.26)	66	1.01 (0.74–1.38)
Beef steak	190,597	283	0.98 (0.84–1.14)	168	0.99 (0.81–1.20)	39	1.12 (0.75–1.67)
Beef soup	340,717	207	1.04 (0.93–1.17)	328	1.06 (0.91–1.23)	79	0.99 (0.74–1.33)
Beef in vegetable soup	137,751	227	1.02 (0.86–1.20)	207	1.06 (0.85–1.32)	29	0.96 (0.61–1.50)
Fried chicken	209,790	280	0.93 (0.79–1.09)	178	0.96 (0.77–1.20)	41	1.07 (0.70–1.62)

HR, (hazard ratios) are for highest vs. lowest intake of meat, CVD, cardiovascular disease. Models were adjusted for baseline age, marital status, education, income, job, smoking, drinking, regular physical exercise, total energy intake, total meat intake, history of chronic diseases, body mass index, and menopausal status in women. HR, hazard ratio. 95% confidence intervals, sample sizes and person-years are shown in Supplementary Tables 5, 6.

## 4. Discussion

Although the associations of meat intake with mortality, particularly red meat and processed meat are well recognized in Western populations where meat intake is a dominant part of the diet, the relationship between meat intake and mortality in Asian populations that have low absolute meat intake and diverse meat preparation methods is not well characterized. In this large cohort from an Asian population, the novel aspect is the evaluation of meat intake by type, and preparation method in relation to total and cause-specific mortality.

High intake of processed red meat was positively associated with all-cause mortality in men and women, and high intake of organ meat was positively associated with all cause and cancer specific mortality in women. However, moderate intake of pork belly was inversely associated with all-cause mortality, but high intake was positively associated with a high risk of CVD mortality in men. Moreover, low beef intake was inversely associated with CVD mortality in men. Considering different meat dishes, roasted pork ribs and pork belly were associated with an increased risk of cancer mortality in women. No evidence of association was reported with white meat.

Total meat was not associated with all-cause mortality in both men and women in line with pooled data (13) and previous meta-analyses in Asian populations (7, 11). Thus, it has been suggested that meat types should be treated separately when analyzing their health effects (7, 11). Total red meat intake was inversely associated with all-cause mortality using pooled data from Asian studies (13) but was not associated with mortality in our analysis. In the former, the study did not distinguish processed from unprocessed red meat, and failed to account for differences in preparation methods. In previous studies, the low intake of red meat and processed meat in Asia has been suggested as one of the possible explanations for null or inverse associations between meat intake and health outcomes (11, 26, 27), with some authors arguing that the consumption of processed and red meat in Korea is not a cause for health concerns. Yet, even in the Adventist community with low intake of meat, the intake of red and processed meat was associated with all-cause and CVD mortality (4). In the current study using data from the Korean population with low meat intake, processed red meat was positively associated with all-cause mortality suggesting that processed meat may have detrimental health effects even at lower intake amounts. The positive association of processed meat with all-cause and CVD mortality is widely reported in Western populations (4, 6, 10–12, 27, 28). Processed, but not unprocessed red meat intake was associated with total and CVD mortality using data from 21 countries (9), and with all-cause mortality in two meta-analyses (27, 28).

Iron mutagens generated by high-temperature cooking (29, 30), N-nitroso compounds formed in processed meat and endogenously from heme iron (31, 32) are some of the mechanisms that may explain the detrimental health impacts of processed red meat. In animal studies, metabolism by intestinal microbiota of dietary L-carnitine-a trimethylamine abundant in red meat, also produces Trimethylamine Oxide (TMAO) and accelerates atherosclerosis (33). Processed red meats are also high in food additives especially nitrite/nitrates. Nitrates/nitrites in processed meat mediated up to 72% of the association between processed meat intake and mortality (10).

Moderate intake of pork belly was inversely associated with all-cause mortality, and low intake of beef appeared to be protective against CVD mortality in men. These associations could be attributed to beef preparation methods in the Korean population. Meat is consumed roasted or in soup or stews, preferably with soybean paste. Beef is popularly prepared as “Bulgogi”- grilled beef flavored with garlic, onions, soy sauce, and sesame oil; and pork is prepared by steaming, stewing, boiling, or smoking (16). In Korea, pork is the most consumed red meat and there is a unique preference for pork belly (“*Sam-gyeop-sal*”) among Korean consumers (34). In Western countries, pork belly is primarily cured and processed as bacon, but consumers in South Korea favor grilled or roasted bellies rather than cured or processed bacon (16). Thus, pork belly consumed in South Korea is lower in saturated fat than that consumed in Western countries due to different preparation methods (34). It should also be noted that consumption of pork belly and beef are common at social gatherings among the middle- and high-income class in the Korean society. Thus, the consumption of these meats could reflect high socio-economic status and high social capital in the Korean population. Our results suggest that moderate intake of unprocessed pork belly and beef may offer protective benefits against premature mortality.

When we considered meat preparation methods, the intake of roasted pork increased the risk of cancer mortality in women. Iron mutagens generated by high-temperature cooking are possible explanations of these associations (29, 30). In addition, direct frying or grilling of meat generates mutagenic Heterocyclic amines (HCAs) and polycyclic aromatic hydrocarbons (PAHs) (30) which have been linked to the development of several cancers (35–40).

Results from a recent meta-analysis reported a 6% reduction in all-cause mortality with high intake of white meat and a null association with CVD-mortality in Asian populations (7). Furthermore, a recent study from Japan reported a reduced risk of cancer mortality with increased intake of chicken in men (14). In the NIH-AARP Diet and Health Study, white meat intake was inversely associated with all-cause and cause-specific mortality (10). Poultry meat contains more unsaturated fat, and has a lower content of saturated fatty acids, heme iron, glycotoxins and sodium, which may be involved in oxidative stress, and atherosclerosis (10, 31, 32, 41). Unlike red meat, white meat does not form N-nitroso compounds (42, 43), and it has been suggested that this could possibly explain the inverse association between white meat intake and mortality risk. Our finding that white meat intake is not associated with mortality does not agree with pooled data from Asian studies that showed that chicken intake was inversely associated with reduced risk of all-cause mortality in men (13), but agrees with several studies that reported a null association between white meat intake and all-cause or CVD mortality (6, 9, 11, 12).

Several limitations should be considered while interpreting these findings. The observational study design precludes confirmation of causal relationships. The inclusion of old adults limits generalizability of our findings to young individuals. The possibility of residual confounding cannot be ruled out even though we adjusted for multiple confounders. We relied on a single dietary assessment at recruitment, and changes in meat intake over time were not evaluated. Nevertheless, we conducted sensitivity analyses by excluding participants with shorter follow-up durations. Dietary data were self-reported, which could have introduced measurement error and biased our results toward the null.

The main strengths of our study include the use of a large sample size from a population-based survey which increases generalizability to the Korean population, the comprehensive evaluation of meat intake by type, cooking and degree of processing in a less-studied population with low meat intake, the prospective study design, adjustment for potential confounding variables, use of a validated SQFFQ, and conducting several sensitivity analyses.

## 5. Conclusion

This study highlighted the unique features of meat consumption patterns in the Korean population, and that the type of red meat, and the preparation methods should be considered in future studies and in designing public health guidelines pertaining to meat intake in this population. The results suggested that processed red meat increased mortality risk from all-causes in men and women, and high intake of organ meat is positively associated with all-cause and cancer specific mortality in women. However, moderate intake of pork belly was inversely associated with all-cause mortality, and low beef intake was inversely associated with CVD mortality in men. Considering different meat dishes, roasted pork ribs and pork belly were associated with increased risk of cancer in women. No evidence of association was reported with white meat.

## Data availability statement

The datasets presented in this article are not readily available because the dataset used for the analysis in this study is maintained and managed by the Division of Population Health Research at the National Institute of Health, Korea Centers for Disease Control and Prevention. The Health Examinees Study dataset has been merged with the cancer registry data provided by National Cancer Center of Korea in a collaborative agreement. It contains some personal data that may potentially be sensitive to the patients, even though researchers are provided with an anonymized dataset that excludes resident registration numbers. Other researchers may request access to the data by contacting the following individuals at the Division of Population Health Research, National Institute of Health, Korea Centers for Disease Control and Prevention: Requests to access the datasets should be directed to Dr. Kyoungho Lee (khlee3789@korea.kr).

## Ethics statement

The studies involving human participants were reviewed and approved by the Ethics Committee of the Korean Health and Genomic Study of the Korean National Institute of Health and the Institutional Review Boards of all participating hospitals (IRB no. E-1503-103- 657). The patients/participants provided their written informed consent to participate in this study.

## Author contributions

AK, S-AL, and DK designed the research. AK conducted the research, analyzed the data, and wrote the paper. S-AL and DK had primary responsibility for final content. All authors approved the final version of the manuscript.
