# Supplementary material for: Total and cause-specific mortality associated with meat intake in a large cohort study in Korea
Source: Front Nutr. 2023 Mar 13;10:1138102. doi: 10.3389/fnut.2023.1138102 (PMC10043978; doi:10.3389/fnut.2023.1138102)
Supplement: Supplementary file 1 [file Table_1.docx]

Table 1. Dietary intake according to quintiles of total meat intake

|  | **Quintiles of total meat intake** | | | | |
| --- | --- | --- | --- | --- | --- |
| **Men** | **Q1** | **Q2** | **Q3** | **Q4** | **Q5** |
| Mean energy intake, Kcal/d | 1569.8± 4.68 | 1682.1± 4.61 | 1801.6± 4.61 | 1941.8± 4.63 | 2214.9± 4.64 |
| Vegetable | 100.5± 0.98 | 97.7± 0.95 | 101.4± 0.94 | 104.6± 0.94 | 111.8± 1.01 |
| Fish | 31.7± 0.37 | 31.8± 0.35 | 34.1± 0.35 | 36.9± 0.35 | 42.3± 0.38 |
| Seafood | 7.9± 0.13 | 8.7± 0.13 | 9.4± 0.12 | 10.2± 0.12 | 12.0± 0.13 |
| Total meat | 12.7 (7.5-16.9) | 28.2 (24.2-31.9) | 44.3 (40-49) | 68.7 (60.9-78.3) | 123.1 (103-161.3) |
| Total RM | 11.5 (6.5-15) | 25.2 (21.7-29.1) | 40.6 (36.3-45.3) | 63.8 (55.8-73) | 115.6 (96.3-152.1) |
| Unprocessed RM | 11.2 (6.3-15) | 25 (21.3-28.6) | 40 (35.6-44.6) | 62.6 (54.9-72) | 113.7 (94.5-149.9) |
| Processed RM | 0.0 (0-0) | 0.0 (0-0) | 0.0 (0-0.7) | 0.0 (0-1.3) | 0.0 (0-2) |
| Total white meat | 1.3 (0-2.5) | 2.5 (1.3-3.1) | 2.5 (2.5-6.3) | 3.8 (2.5-6.3) | 6.3 (2.5-16.1) |
| Total beef | 2.9 (0-5) | 8.1 (4.6-10.8) | 11.3 (6.7-17.1) | 18.6 (9.6-29) | 40.5 (21.4-65.7) |
| Total pork | 6.3 (2.6-10.4) | 15.6 (12.1-19.8) | 26.9 (20-32.5) | 39.6 (29.3-50.5) | 70.3 (48.8-89.4) |
| Fried chicken | 0.1 (0-0.3) | 0.3 (0.1-0.4) | 0.3 (0.3-0.7) | 0.4 (0.3-0.7) | 0.7 (0.3-1.8) |
| Organ meat | 0.0 (0-0.1) | 0.1 (0.1-2.9) | 1.5 (0.1-3.1) | 2.9 (0.1-3.9) | 3.1 (0.4-7.7) |
| Beef with soup | 1.7 (0-3.3) | 3.3 (1.7-5) | 4.2 (2.5-8.3) | 8.3 (3.3-14.9) | 14.9 (5.8-50) |
| **Women** |  |  |  |  |  |
| Mean energy intake, Kcal/d | 1423.5± 3.62 | 1528± 3.57 | 1634.5± 3.56 | 1766.8± 3.58 | 2044.3± 3.59 |
| Vegetable | 113.4± 0.78 | 109.9± 0.76 | 111.4± 0.75 | 112.8± 0.76 | 119.8± 0.8 |
| Fish | 31.1± 0.26 | 30.5± 0.26 | 32.1± 0.26 | 34.5± 0.26 | 39.3± 0.27 |
| Seafood | 8.3± 0.1 | 8.5± 0.1 | 9.1± 0.1 | 10.1± 0.1 | 11.6± 0.1 |
| Total meat | 6.3 (2.5-9.4) | 17.5 (15-20.4) | 29.8 (26.3-33.8) | 48.8 (43.1-55.7) | 94.3 (77.3-125.4) |
| Total RM | 5 (1.7-8.3) | 15.4 (12.9-18.3) | 26.7 (23.3-30.7) | 44.4 (38.9-51.2) | 87.2 (71.4-117.1) |
| Unprocessed RM | 5 (1.7-8.1) | 15 (12.7-18.1) | 26.2 (22.6-30.1) | 43.6 (38.1-50.2) | 85.5 (70-115) |
| Processed RM | 0.0 (0-0) | 0.0 (0-0) | 0.0 (0-0.7) | 0.0 (0-1.3) | 0.0 (0-1.7) |
| Total white meat | 0 (0-1.3) | 2.5 (0-2.5) | 2.5 (1.3-3.8) | 2.5 (2.5-6.3) | 6.3 (2.5-9.4) |
| Total beef | 1.5 (0-3.3) | 5.0 (2.3-7.9) | 8.3 (5-12.5) | 14.2 (8.3-21.5) | 33.8 (15.7-55) |
| Total pork | 2.5 (0-5) | 10 (6.7-12.5) | 17 (12.5-21.3) | 28.5 (19.8-35.8) | 54.7 (34.6-74.4) |
| Fried chicken | 0 (0-0.1) | 0.3 (0-0.3) | 0.3 (0.1-0.4) | 0.3 (0.3-0.7) | 0.7 (0.3-1.1) |
| Organ meat | 0 (0-0.1) | 0.1 (0-0.4) | 0.1 (0.0-2.9) | 0.4 (0.0-3.1) | 0.9 (0.1-3.9) |
| Beef with soup | 0 (0-1.7) | 1.7 (0-3.3) | 3.3 (1.7-5.4) | 4.2 (1.7-8.3) | 8.3 (3.3-26.7) |

Values are mean±standard error or median (Q1-Q3). RM, red meat

Table 2. Median intake of total meat and meat sub-types in men and women

|  | **Meat intake, grams/day** |  |
| --- | --- | --- |
| **Item** | **Men** | **Women** |
| Total meat | 44.3 | 29.7 |
| Total RM | 40.4 | 26.5 |
| Unprocessed RM | 39.6 | 25.8 |
| Processed RM* | 0.7 | 0.0 |
| Total pork | 24.3 | 14.6 |
| Pork belly | 12.5 | 5.0 |
| Roasted pork | 5.0 | 5.0 |
| Braised pork | 1.7 | 0.8 |
| Total beef | 10.4 | 7.9 |
| Beef soup | 1.7 | 0 |
| Beef soup in vegetables | 1.7 | 1.7 |
| White meat | 2.2 | 2.2 |
| Fried chicken | 0.3 | 0.3 |
| Organ meat | 0.4 | 0.1 |

*75^th^ percentile. RM, red meat

Table 3. All-cause, cancer and CVD mortality associated with meat intake by type of meat in men.

|  |  |  | **Q1** |  |  | **Q2** |  |  | **Q3** |  |  | **Q4** |  |  | **Q5** |
| --- | --- | --- | --- | --- | --- | --- | --- | --- | --- | --- | --- | --- | --- | --- | --- |
| **All-cause mortality** | **n** | **PY** | **HR (95% CI)** | **n** | **PY** | **HR (95% CI)** | **n** | **PY** | **HR (95% CI)** | **n** | **PY** | **HR (95% CI)** | **n** | **PY** | **HR (95% CI)** |
| Total meat | 566 | 81684 | 1.00 | 424 | 81982 | 0.92 (0.81-1.05) | 372 | 82432 | 0.94 (0.82-1.08) | 396 | 81718 | 1.09 (0.95-1.26) | 308 | 82048 | 0.91 (0.77-1.07) |
| Total RM | 557 | 81638 | 1.00 | 430 | 81947 | 0.95 (0.83-1.08) | 368 | 82375 | 0.93 (0.81-1.07) | 401 | 81770 | 1.10 (0.95-1.26) | 310 | 82133 | 0.90 (0.76-1.05) |
| Unprocessed RM | 559 | 81424 | 1.00 | 426 | 82189 | 0.91 (0.8-1.03) | 361 | 82220 | 0.90 (0.78-1.03) | 403 | 81871 | 1.08 (0.94-1.24) | 317 | 82159 | 0.89 (0.76-1.05) |
| Processed RM | 1618 | 287049 | 1.00 | 90 | 29556 | 0.81 (0.65-1.01) | **358** | **93258** | **1.21 (1.07-1.37)** |  |  |  |  |  |  |
| Total pork | 571 | 81428 | 1.00 | 444 | 81752 | 0.95 (0.83-1.07) | 395 | 82118 | 0.99 (0.86-1.13) | 324 | 82260 | 0.87 (0.75-1) | 332 | 82305 | 0.98 (0.84-1.15) |
| Pork belly | 478 | 67738 | 1.00 | 549 | 96604 | 0.98 (0.86-1.11) | 483 | 105192 | 0.90 (0.79-1.03) | 122 | 35331 | 0.76 (0.62-0.93) | 434 | 104998 | 0.89 (0.77-1.03) |
| Beef | 555 | 86057 | 1.00 | 376 | 73688 | 0.95 (0.83-1.08) | 419 | 87237 | 1.01 (0.88-1.15) | 337 | 81641 | 0.96 (0.83-1.11) | 379 | 81242 | 1.08 (0.93-1.24) |
| Organ meat | 634 | 98844 | 1.00 | 573 | 108951 | 0.97 (0.86-1.08) | 478 | 101978 | 1.01 (0.89-1.14) | 381 | 100090 | 1.02 (0.89-1.17) |  |  |  |
| White meat | 590 | 87833 | 1.00 | 196 | 36908 | 1.02 (0.87-1.20) | 657 | 126216 | 1.04 (0.92-1.16) | 448 | 106992 | 1.04 (0.91-1.19) | 175 | 51914 | 1.03 (0.86-1.23) |
| **Cancer mortality** |  |  |  |  |  |  |  |  |  |  |  |  |  |  |  |
| Total meat | 251 | 81684 | 1.00 | 199 | 81982 | 0.99 (0.82-1.2) | 176 | 82432 | 1.02 (0.83-1.24) | 191 | 81718 | **1.23 (1.01-1.5)** | 135 | 82048 | 0.94 (0.74-1.2) |
| Total RM | 249 | 81638 | 1.00 | 193 | 81947 | 0.97 (0.8-1.17) | 183 | 82375 | 1.07 (0.87-1.3) | 191 | 81770 | 1.23 (1-1.51) | 136 | 82133 | 0.95 (0.75-1.21) |
| Unprocessed RM | 249 | 81424 | 1.00 | 193 | 82189 | 0.94 (0.78-1.14) | 179 | 82220 | 1.02 (0.84-1.25) | 192 | 81871 | 1.21 (0.99-1.49) | 139 | 82159 | 0.95 (0.74-1.2) |
| Processed RM | 755 | 287049 | 1.00 | 45 | 29556 | 0.86 (0.63-1.17) | 152 | **93258** | 1.12 (0.93-1.34) |  |  |  |  |  |  |
| Total pork | 257 | 81428 | 1.00 | 212 | 81752 | 1.02 (0.85-1.23) | 186 | 82118 | 1.07 (0.88-1.3) | 143 | 82260 | 0.89 (0.72-1.11) | 154 | 82305 | 1.09 (0.87-1.37) |
| Pork belly | 233 | 67738 | 1.00 | 74 | 96604 | 1.07 (0.89-1.3) | 192 | 105192 | 1.03 (0.85-1.26) | 133 | 35331 | 0.83 (0.61-1.12) | 136 | 104998 | 1.06 (0.86-1.31) |
| Beef | 244 | 86057 | 1.00 | 183 | 73688 | 1.03 (0.85-1.25) | 197 | 87237 | 1.07 (0.88-1.3) | 149 | 81641 | 0.96 (0.78-1.20) | 179 | 81242 | 1.18 (0.96-1.47) |
| Organ meat | 300 | 98844 | 1.00 | 258 | 108951 | 0.91 (0.77-1.08) | 216 | 101978 | 0.95 (0.79-1.14) | 178 | 100090 | 1.00 (0.82-1.22) |  |  |  |
| White meat | 276 | 87833 | 1.00 | 101 | 36908 | 1.13 (0.9-1.42) | 310 | 126216 | 1.03 (0.87-1.22) | 190 | 106992 | 0.93 (0.76-1.13) | 75 | 51914 | 0.93 (0.71-1.23) |
| **CVD mortality** |  |  |  |  |  |  |  |  |  |  |  |  |  |  |  |
| Total meat | 86 | 81684 | 1.00 | 63 | 81982 | 0.90 (0.65-1.25) | 65 | 82432 | 1.07 (0.77-1.5) | 69 | 81718 | 1.21 (0.86-1.7) | 50 | 82048 | 0.91 (0.61-1.35) |
| Total RM | 86 | 81638 | 1.00 | 65 | 81947 | 0.92 (0.66-1.27) | 61 | 82375 | 0.97 (0.68-1.36) | 70 | 81770 | 1.14 (0.81-1.61) | 51 | 82133 | 0.82 (0.55-1.22) |
| Unprocessed RM | 86 | 81424 | 1.00 | 66 | 82189 | 0.90 (0.65-1.25) | 58 | 82220 | 0.90 (0.63-1.28) | 72 | 81871 | 1.15 (0.81-1.61) | 51 | 82159 | 0.79 (0.53-1.18) |
| Processed RM | 262 | 287049 | 1.00 | 11 | 29556 | 0.62 (0.34-1.15) | 60 | **93258** | 1.21 (0.9-1.63) |  |  |  |  |  |  |
| Total pork | 91 | 81428 | 1.00 | 75 | 81752 | 0.97 (0.71-1.33) | 59 | 82118 | 0.88 (0.62-1.24) | 54 | 82260 | 0.82 (0.57-1.18) | 54 | 82305 | 0.85 (0.58-1.25) |
| Pork belly | 76 | 67738 | 1.00 | 93 | 96604 | 1.01 (0.74-1.38) | 75 | 105192 | 0.85 (0.61-1.18) | 19 | 35331 | 0.67 (0.4-1.13) | 70 | 104998 | 0.79 (0.55-1.14) |
| Beef | 97 | 86057 | 1.00 | **40** | 73688 | **0.58 (0.40-0.84)** | 68 | 87237 | 0.93 (0.68-1.29) | 68 | 81641 | 1.07 (0.77-1.5) | 60 | 81242 | 0.88 (0.62-1.26) |
| Organ meat | 98 | 98844 | 1.00 | 95 | 108951 | 1.03 (0.77-1.37) | 83 | 101978 | 1.13 (0.83-1.52) | 57 | 100090 | 0.97 (0.68-1.37) |  |  |  |
| White meat | 94 | 87833 | 1.00 | 29 | 36908 | 0.96 (0.63-1.46) | 95 | 126216 | 0.95 (0.71-1.27) | 77 | 106992 | 1.14 (0.83-1.57) | 38 | 51914 | 1.45 (0.96-2.19) |

Numbers are HR (95% CI) adjusted for age, education, income, area of residence, marital status, smoking, drinking, regular exercise, BMI, disease score, and total red or white meat intake (in individual meat models). RM. Red meat.

Table 4. All-cause, cancer and CVD mortality associated with meat intake by type of meat in women.

|  | **Q1** | | |  |  | **Q2** |  |  | **Q3** |  |  | **Q4** |  |  | **Q5** |
| --- | --- | --- | --- | --- | --- | --- | --- | --- | --- | --- | --- | --- | --- | --- | --- |
| **All-cause mortality** | **n** | **PY** | **HR (95% CI)** | **n** | **PY** | **HR (95% CI)** | **n** | **PY** | **HR (95% CI)** | **n** | **PY** |  | **n** | **PY** |  |
| Total meat | 395 | 159362 | 0.9 (0.77-1.05) | 278 | 159584 | 0.9 (0.77-1.05) | 264 | 159535 | 0.98 (0.83-1.15) | 228 | 159015 | 0.94 (0.79-1.12) | 223 | 157875 | 1.02 (0.85-1.22) |
| Total RM | 393 | 158699 | 1.00 | 264 | 159723 | 0.86 (0.73-1.01) | 262 | 159940 | 0.99 (0.84-1.16) | 248 | 158861 | 1.04 (0.87-1.23) | 221 | 158148 | 0.99 (0.82-1.2) |
| Unprocessed RM | 393 | 158572 | 1.00 | 268 | 160189 | 0.87 (0.74-1.02) | 257 | 159349 | 0.96 (0.81-1.14) | 240 | 158957 | 1.00 (0.84-1.19) | 230 | 158305 | 1.02 (0.84-1.23) |
| Processed RM | 1136 | 605110 | 1.00 | 62 | 52151 | 1.02 (0.79-1.33) | 190 | 138111 | 1.32 (1.12-1.56) |  |  |  |  |  |  |
| Total pork | 433 | 166149 | 1.00 | 277 | 147421 | 0.94 (0.8-1.1) | 235 | 163939 | 0.81 (0.69-0.96) | 208 | 159122 | 0.87 (0.73-1.04) | 235 | 158740 | 1.12 (0.93-1.34) |
| Pork belly | 433 | 171494 | 1.00 | 133 | 73887 | 0.86 (0.7-1.05) | 353 | 217317 | 0.87 (0.75-1.00) | 217 | 162558 | 0.82 (0.69-0.98) | 252 | 170114 | 1.03 (0.87-1.23) |
| Total beef | 371 | 176406 | 1.00 | 265 | 143096 | 1.07 (0.91-1.25) | 251 | 160128 | 0.97 (0.82-1.14) | 251 | 156623 | 1.05 (0.89-1.25) | 250 | 159118 | 1.04 (0.87-1.24) |
| Organ meat | 618 | 329513 | 1.00 | 36 | 26542 | 0.80 (0.57-1.12) | 411 | 242452 | 1.04 (0.91-1.18) | **323** | **196866** | **1.21 (1.05-1.39)** |  |  |  |
| Total white meat | 522 | 212763 | 1.00 | 150 | 94381 | 0.87 (0.73-1.05) | 366 | 227905 | 0.91 (0.79-1.04) | 70 | 50532 | 0.93 (0.72-1.20) | 280 | 209790 | 0.94 (0.8-1.100) |
| **Cancer mortality** |  |  |  |  |  |  |  |  |  |  |  |  |  |  |  |
| Total meat | 198 | 159362 | 1.00 | 152 | 159584 | 0.93 (0.75-1.16) | 151 | 159535 | 1.04 (0.83-1.29) | 137 | 159015 | 1.03 (0.82-1.3) | 130 | 157875 | 1.05 (0.81-1.34) |
| Total RM | 202 | 158699 | 1.00 | 138 | 159723 | 0.82 (0.66-1.03) | 151 | 159940 | 1.01 (0.81-1.26) | 146 | 158861 | 1.07 (0.85-1.35) | 131 | 158148 | 1.01 (0.78-1.30) |
| Unprocessed red meat | 201 | 158572 | 1.00 | 140 | 160189 | 0.84 (0.67-1.05) | 150 | 159349 | 1.00 (0.80-1.25) | 142 | 158957 | 1.04 (0.82-1.31) | 135 | 158305 | 1.03 (0.8-1.32) |
| Processed red meat | 622 | 605110 | 1.00 | 39 | 52151 | 1.10 (0.79-1.54) | 107 | 138111 | 1.24 (0.99-1.54) |  |  |  |  |  |  |
| Total pork | 221 | 166149 | 1.00 | 149 | 147421 | 0.94 (0.76-1.16) | 138 | 163939 | 0.87 (0.7-1.09) | 120 | 159122 | 0.90 (0.71-1.14) | 140 | 158740 | 1.15 (0.90-1.48) |
| Pork belly | 233 | 171494 | 1.00 | 74 | 73887 | 0.85 (0.65-1.12) | 192 | 217317 | 0.83 (0.68-1.01) | 133 | 162558 | 0.86 (0.69-1.08) | 136 | 170114 | 0.92 (0.73-1.16) |
| Beef | 198 | 176406 | 1.00 | 141 | 143096 | 1.00 (0.80-1.25) | 134 | 160128 | 0.89 (0.71-1.12) | 153 | 156623 | 1.08 (0.86-1.35) | 142 | 159118 | 0.97 (0.77-1.23) |
| Organ meat | 323 | 329513 | 1.00 | 17 | 26542 | 0.71 (0.44-1.16) | 246 | 242452 | 1.15 (0.97-1.37) | **182** | **196866** | **1.24 (1.03-1.50)** |  |  |  |
| Total white meat | 264 | 212763 | 1.00 | 87 | 94381 | 0.96 (0.75-1.23) | 211 | 227905 | 0.97 (0.80-1.17) | 43 | 50532 | 1.06 (0.76-1.47) | 163 | 209790 | 0.98 (0.79-1.21) |
| **CVD mortality** |  |  |  |  |  |  |  |  |  |  |  |  |  |  |  |
| Total meat | 62 | 159362 | 1.00 | 40 | 159584 | 0.94 (0.63-1.4) | 36 | 159535 | 1.04 (0.68-1.58) | 35 | 159015 | 1.17 (0.76-1.81) | 33 | 157875 | 1.28 (0.8-2.06) |
| Total RM | 60 | 158699 | 1.00 | 41 | 159723 | 0.97 (0.65-1.46) | 36 | 159940 | 1.05 (0.68-1.63) | 38 | 158861 | 1.27 (0.81-1.97) | 31 | 158148 | 1.14 (0.69-1.88) |
| Unprocessed red meat | 60 | 158572 | 1.00 | 41 | 160189 | 0.97 (0.64-1.45) | 35 | 159349 | 1.02 (0.65-1.58) | 37 | 158957 | 1.22 (0.78-1.91) | 33 | 158305 | 1.19 (0.73-1.94) |
| Processed red meat | 177 | 605110 | 1.00 | 07 | 52151 | 0.93 (0.43-2) | 22 | 138111 | 1.18 (0.74-1.89) |  |  |  |  |  |  |
| Total pork | 61 | 166149 | 1.00 | 40 | 147421 | 1.08 (0.72-1.63) | 38 | 163939 | 1.10 (0.72-1.68) | 35 | 159122 | 1.33 (0.85-2.08) | 32 | 158740 | 1.46 (0.89-2.38) |
| Pork belly | 59 | 171494 | 1.00 | 15 | 73887 | 0.77 (0.43-1.37) | 55 | 217317 | 1.12 (0.77-1.64) | 31 | 162558 | 1.04 (0.66-1.64) | **46** | 170114 | **1.84 (1.20-2.82)** |
| Beef | 56 | 176406 | 1.00 | 41 | 143096 | 1.20 (0.79-1.8) | 46 | 160128 | 1.34 (0.89-2.01) | 28 | 156623 | 0.90 (0.56-1.44) | 35 | 159118 | 1.09 (0.69-1.73) |
| Organ meat | 99 | 329513 | 1.00 | 04 | 26542 | 0.61 (0.22-1.67) | 52 | 242452 | 0.88 (0.63-1.25) | 51 | **196866** | 1.35 (0.95-1.92) |  |  |  |
| Total white meat | 82 | 212763 | 1.00 | 22 | 94381 | 0.93 (0.58-1.51) | 52 | 227905 | 0.92 (0.64-1.33) | 9 | 50532 | 0.94 (0.47-1.89) | 41 | 209790 | 1.09 (0.72-1.65) |

Numbers are HR (95% CI) adjusted for age, education, income, area of residence, marital status, smoking, drinking, regular exercise, BMI, disease score, reproductive factors, and total red or white meat intake (in individual meat models). RM. Red meat

Table 5. All-cause, cancer and CVD mortality associated with Meat intake by meat preparation method in men

|  |  |  | **Q1** |  |  | **Q2** |  |  | **Q3** |  |  | **Q4** |
| --- | --- | --- | --- | --- | --- | --- | --- | --- | --- | --- | --- | --- |
| **All-cause mortality** | **n** | **PY** | **HR (95% CI)** | **n** | **PY** | **HR (95% CI)** | **n** | **PY** | **HR (95% CI)** | **n** | **PY** | **HR (95% CI)** |
| Roasted pork | 588 | 91956 | 1.00 | 614 | 120445 | 0.95 (0.85-1.06) | 502 | 107944 | 0.99 (0.88-1.12) | 362 | 89519 | 0.95 (0.82-1.09) |
| Braised pork | 1010 | 171143 | 1.00 | 147 | 138427 | 1.03 (0.93-1.14) | 493 | 100294 | 1.06 (0.94-1.19) | 416 |  |  |
| Beef steak | 827 | 138995 | 1.00 | 165 | 35520 | 0.91 (0.77-1.07) | 626 | 129188 | 1.00 (0.9-1.11) | 448 | 106160 | 0.97 (0.86-1.1) |
| Beef soup | 795 | 131795 | 1.00 | 89 | 151453 | 1.01 (0.92-1.12) | 699 | 126615 | 1.00 (0.89-1.13) | 483 | 85649 |  |
| Beef in vegetable soup | 626 | 99498 | 1.00 | 676 | 133444 | 0.99 (0.88-1.10) | 360 | 91272 | 0.9 (0.79-1.03) | 404 |  | 1.01 (0.88-1.15) |
| Fried chicken | 590 | 87833 | 1.00 | 853 | 163124 | 1.03 (0.92-1.15) | 118 | 27561 | 1.05 (0.86-1.29) | 505 | 131345 | 1.02 (0.9-1.17) |
| **Cancer mortality** |  |  |  |  |  |  |  |  |  |  |  |  |
| Roasted pork | 283 | 91956 | 1.00 | 274 | 120445 | 0.88 (0.74-1.04) | 232 | 107944 | 0.96 (0.81-1.15) | 163 | 89519 | 0.92 (0.75-1.13) |
| Braised pork | 465 | 171143 | 1.00 | 290 | 138427 | 1.01 (0.87-1.17) | 197 | 100294 | 1.12 (0.94-1.33) |  |  |  |
| Beef steak | 370 | 138995 | 1.00 | 76 | 35520 | 0.92 (0.72-1.18) | 285 | 129188 | 0.99 (0.85-1.16) | 221 | 106160 | 1.06 (0.89-1.27) |
| Beef soup | 380 | 131795 | 1.00 | 347 | 151453 | 0.95 (0.82-1.1) | 225 | 126615 | 0.93 (0.78-1.11) |  | 85649 |  |
| Beef in vegetable soup | 299 | 99498 | 1.00 | 315 | 133444 | 0.96 (0.82-1.12) | 155 | 91272 | 0.82 (0.67-0.99) | 183 |  | 0.99 (0.81-1.2) |
| Fried chicken | 276 | 87833 | 1.00 | 411 | 163124 | 1.06 (0.9-1.24) | 43 | 27561 | 0.82 (0.59-1.13) | 222 | 131345 | 0.96 (0.79-1.17) |
| **CVD mortality** |  |  |  |  |  |  |  |  |  |  |  |  |
| Roasted pork | 96 | 91956 | 1.00 | 109 | 120445 | 1.02 (0.77-1.34) | 71 | 107944 | 0.82 (0.6-1.13) | 57 | 89519 | 0.81 (0.57-1.14) |
| Braised pork | 157 | 171143 | 1.00 | 107 | 138427 | 1.10 (0.86-1.41) | 69 | 100294 | 1.05 (0.78-1.42) |  |  |  |
| Beef steak | 134 | 138995 | 1.00 | 21 | 35520 | 0.73 (0.46-1.17) | 112 | 129188 | 1.10 (0.85-1.42) | 66 | 106160 | 0.83 (0.6-1.13) |
| Beef soup | 125 | 131795 | 1.00 | 123 | 151453 | 1.04 (0.81-1.34) | 85 | 126615 | 1.01 (0.76-1.36) |  | 85649 |  |
| Beef in vegetable soup | 88 | 99498 | 1.00 | 113 | 133444 | 1.17 (0.88-1.55) | 63 | 91272 | 1.09 (0.78-1.52) | 69 |  | 1.12 (0.8-1.57) |
| Fried chicken | 94 | 87833 | 1.00 | 124 | 163124 | 0.96 (0.73-1.26) | 21 | 27561 | 1.22 (0.75-1.99) | 94 | 131345 | 1.25 (0.91-1.72) |

Numbers are HR (95% CI) adjusted for age, education, income, area of residence, marital status, smoking, drinking, regular exercise, BMI, disease score, total energy and total red or white meat intake.

Table 6. All-cause, cancer and CVD mortality associated with Meat intake by meat preparation method in women

|  |  |  | **Q1** |  |  | **Q2** |  |  | **Q3** |  |  | **Q4** |
| --- | --- | --- | --- | --- | --- | --- | --- | --- | --- | --- | --- | --- |
| **All-cause mortality** | **n** | **PY** | **HR (95% CI)** | **n** | **PY** | **HR (95% CI)** | **n** | **PY** | **HR (95% CI)** | **n** | **PY** | **HR (95% CI)** |
| Roasted pork | 558 | 262695 | 1.00 | 138 | 85186 | 0.91 (0.75-1.1) | 330 | 206717 | 0.94 (0.82-1.08) | 362 | 240774 | 1.10 (0.95-1.26) |
| Braised pork | 764 | 386277 | 1.00 | 582 | 409094 | 1.01 (0.90-1.12) |  |  |  |  |  |  |
| Beef steak | 624 | 303237 | 1.00 | 144 | 87269 | 0.96 (0.8-1.16) | 337 | 214267 | 0.93 (0.81-1.06) | 283 | 190597 | 0.98 (0.84-1.14) |
| Beef soup | 744 | 409597 | 1.00 | 602 | 385775 | 0.98 (0.88-1.10) |  |  |  |  |  |  |
| Beef in vegetable soup | 481 | 245810 | 1.00 | 70 | 52724 | 0.82 (0.64-1.06) | 610 | 359085 | 1.06 (0.94-1.19) | 227 | 137751 | 1.02 (0.86-1.2) |
| Fried chicken | 522 | 212763 | 1.00 | 150 | 94381 | 0.87 (0.72-1.05) | 436 | 278437 | 0.91 (0.79-1.04) | 280 | 209790 | 0.93 (0.79-1.09) |
| **Cancer mortality** |  |  |  |  |  |  |  |  |  |  |  |  |
| Roasted pork | 278 | 262695 | 1.00 | 80 | 85186 | 1.04 (0.81-1.33) | 188 | 206717 | 1.04 (0.87-1.26) | **222** | 240774 | **1.26 (1.05-1.52)** |
| Braised pork | 412 | 386277 | 1.00 | 72 | 81703 | 1.04 (0.81-1.34) | 284 | 327391 | 1.08 (0.92-1.26) |  |  |  |
| Beef steak | 331 | 303237 | 1.00 | 79 | 87269 | 0.95 (0.74-1.22) | 190 | 214267 | 0.92 (0.77-1.11) | 168 | 190597 | 0.99 (0.81-1.2) |
| Beef soup | 415 | 409597 | 1.00 | 25 | 45057 | 0.64 (0.42-0.95) | 328 | 340717 | 1.06 (0.91-1.23) |  |  |  |
| Beef in vegetable soup | 258 | 245810 | 1.00 | 33 | 52724 | 0.70 (0.49-1.01) | 341 | 359085 | 1.05 (0.89-1.23) | 136 | 137751 | 1.06 (0.85-1.32) |
| Fried chicken | 264 | 212763 | 1.00 | 87 | 94381 | 0.96 (0.75-1.22) | 254 | 278437 | 0.98 (0.82-1.18) | 163 | 209790 | 0.96 (0.77-1.2) |
| **CVD mortality** |  |  |  |  |  |  |  |  |  |  |  |  |
| Roasted pork | 91 | 262695 | 1.00 | 17 | 85186 | 0.74 (0.44-1.24) | 45 | 206717 | 0.87 (0.61-1.25) | 53 | 240774 | 1.16 (0.81-1.67) |
| Braised pork | 127 | 386277 | 1.00 | 13 | 81703 | 0.76 (0.43-1.35) | 66 | 327391 | 1.01 (0.74-1.38) |  |  |  |
| Beef steak | 94 | 303237 | 1.00 | 23 | 87269 | 1.18 (0.74-1.88) | 50 | 214267 | 1.06 (0.74-1.5) | 39 | 190597 | 1.12 (0.75-1.67) |
| Beef soup | 119 | 409597 | 1.00 | 08 | 45057 | 0.83 (0.40-1.7) | 79 | 340717 | 0.99 (0.74-1.33) |  |  |  |
| Beef in vegetable soup | 70 | 245810 | 1.00 | 7 | 52724 | 0.64 (0.29-1.39) | 100 | 359085 | 1.34 (0.98-1.82) | 29 | 137751 | 0.96 (0.61-1.5) |
| Fried chicken | 82 | 212763 | 1.00 | 22 | 94381 | 0.93 (0.57-1.49) | 61 | 278437 | 0.92 (0.65-1.3) | 41 | 209790 | 1.07 (0.70-1.62) |

Numbers are HR (95% CI) adjusted for age, education, income, area of residence, marital status, smoking, drinking, regular exercise, BMI, disease score, reproductive factors (women), total energy and total red or white meat intake.

Table 7. Sensitivity analysis in men

| **All-cause mortality** | n^a^ | PY^a^ | HR (95% CI)^a^ | HR (95% CI)^b^ | HR (95% CI)^c^ |
| --- | --- | --- | --- | --- | --- |
| Total meat | 292 | 82039 | 0.91 (0.77-1.07) | 0.89 (0.76-1.04) | 0.87 (0.74-1.02) |
| Total RM | 295 | 82125 | 0.88 (0.75-1.04) | 0.87 (0.74-1.02) | 0.85 (0.72-1) |
| Unprocessed RM | 302 | 82151 | 0.88 (0.75-1.03) | 0.86 (0.74-1.01) | 0.84 (0.72-0.99) |
| Processed RM | 342 | 93250 | **1.21 (1.07-1.37)** | **1.21 (1.07-1.37)** | **1.20 (1.07-1.36)** |
| Pork | 316 | 82253 | 0.99 (0.84-1.15) | 0.94 (0.81-1.1) | 0.93 (0.8-1.09) |
| Pork belly | 417 | 104989 | 0.92 (0.8-1.06) | 0.86 (0.75-0.99) | 0.85 (0.74-0.98) |
| Beef | 365 | 81234 | 1.08 (0.93-1.25) | 1.07 (0.92-1.23) | 1.04 (0.9-1.21) |
| Visceral meat | 367 | 100082 | 1.03 (0.89-1.18) | 1.00 (0.87-1.15) | 0.98 (0.85-1.13) |
| White meat | 163 | 51906.7 | 1.00 (0.83-1.2) | 1.02 (0.85-1.22) | 1.00 (0.83-1.2) |
| **Cancer mortality** |  |  |  |  |  |
| Total meat | 127 | 82039 | 0.94 (0.73-1.19) | 0.91 (0.72-1.16) | 0.88 (0.69-1.12) |
| Total RM | 129 | 82125 | 0.95 (0.74-1.21) | 0.91 (0.72-1.16) | 0.88 (0.69-1.13) |
| Unprocessed RM | 132 | 82151 | 0.94 (0.74-1.21) | 0.91 (0.72-1.16) | 0.88 (0.69-1.12) |
| Processed red meat | 147 | 93250 | 1.13 (0.94-1.36) | 1.12 (0.93-1.34) | 1.10 (0.92-1.33) |
| Pork | 147 | 82253 | 1.11 (0.88-1.41) | 1.05 (0.84-1.31) | 1.03 (0.82-1.29) |
| Pork belly | 199 | 104989 | 1.11 (0.89-1.37) | 1.01 (0.82-1.25) | 1.00 (0.81-1.23) |
| Beef | 171 | 81234 | 1.19 (0.96-1.47) | 1.17 (0.95-1.44) | 1.12 (0.91-1.4) |
| Visceral meat | 173 | 100082 | 1.03 (0.84-1.26) | 1.00 (0.82-1.22) | 0.98 (0.8-1.20) |
| White meat | 70 | 51906.7 | 0.93 (0.70-1.23) | 0.95 (0.72-1.25) | 0.92 (0.7-1.21) |
| **CVD mortality** |  |  |  |  |  |
| Total meat | 47 | 82039 | 0.90 (0.60-1.35) | 0.90 (0.61-1.34) | 0.92 (0.61-1.37) |
| Total RM | 48 | 82125 | 0.80 (0.53-1.20) | 0.80 (0.54-1.19) | 0.82 (0.54-1.23) |
| Unprocessed RM | 48 | 82151 | 0.78 (0.52-1.17) | 0.78 (0.52-1.16) | 0.79 (0.53-1.19) |
| Processed RM | 59 | 93250 | 1.25 (0.92-1.68) | 1.20 (0.89-1.61) | 1.21 (0.90-1.63) |
| Pork | 51 | 82253 | 0.86 (0.58-1.27) | 0.84 (0.58-1.23) | 0.84 (0.58-1.24) |
| Pork belly | 66 | 104989 | 0.80 (0.56-1.15) | 0.79 (0.56-1.12) | 0.79 (0.55-1.12) |
| Beef | 59 | 81234 | 0.92 (0.64-1.31) | 0.87 (0.61-1.23) | 0.90 (0.63-1.3) |
| Visceral meat | 55 | 100082 | 0.93 (0.65-1.33) | 0.91 (0.64-1.3) | 0.92 (0.64-1.31) |
| White meat | 35 | 51906.7 | 1.42 (0.93-2.16) | 1.46 (0.97-2.21) | 1.49 (0.98-2.25) |

HR (95% CI) are for highest vs lowest percentiles of meat intake.

a excluding the first 1 year of follow-up

b Adjusted for alcohol intake expressed in grams/day

c Further adjusted for intake of vegetables, fish, sea food and legumes

PY, person-years; RM, red meat

Table 8. Sensitivity analysis in women

| **All-cause mortality** | n^a^ | PY^a^ | HR (95% CI)^a^ | HR (95% CI)^b^ | HR (95% CI)^c^ |
| --- | --- | --- | --- | --- | --- |
| Total meat | 214 | 157869 | 1.03 (0.84-1.25) | 1.02 (0.84-1.23) | 1.02 (0.84-1.24) |
| Total RM | 213 | 158143 | 0.99 (0.81-1.19) | 0.97 (0.8-1.17) | 0.97 (0.80-1.18) |
| Unprocessed RM | 223 | 158300 | 1.02 (0.84-1.23) | 0.99 (0.83-1.2) | 1.00 (0.83-1.20) |
| Processed RM | 181 | 138105 | **1.30 (1.10-1.54)** | **1.31 (1.11-1.54)** | **1.31 (1.11-1.54)** |
| Pork | 224 | 158733 | 1.10 (0.91-1.32) | 1.09 (0.91-1.3) | 1.09 (0.91-1.31) |
| Pork belly | 241 | 170108 | 1.02 (0.86-1.22) | 1.01 (0.85-1.19) | 1.01 (0.85-1.19) |
| Beef | 242 | 159112 | 1.03 (0.86-1.23) | 1.02 (0.86-1.22) | 1.03 (0.86-1.23) |
| Visceral meat | 308 | 196857 | **1.20 (1.04-1.38)** | **1.20 (1.04-1.38)** | **1.21 (1.05-1.40)** |
| White meat | 268 | 209783 | 0.90 (0.77-1.06) | 0.91 (0.78-1.06) | 0.91 (0.78-1.06) |
| **Cancer mortality** |  |  |  |  |  |
| Total meat | 127 | 157869 | 1.04 (0.80-1.35) | 1.05 (0.81-1.36) | 1.03 (0.79-1.34) |
| Total RM | 129 | 158143 | 0.99 (0.77-1.28) | 1.00 (0.78-1.28) | 0.98 (0.76-1.27) |
| Unprocessed RM | 134 | 158300 | 1.03 (0.8-1.32) | 1.02 (0.80-1.31) | 1.00 (0.78-1.29) |
| Processed RM | 104 | 138105 | 1.22 (0.98-1.52) | 1.23 (0.99-1.53) | 1.23 (0.99-1.53) |
| Pork | 118 | 158733 | 1.15 (0.9-1.46) | 1.15 (0.90-1.46) | 1.15 (0.90-1.46) |
| Pork belly | 134 | 170108 | 0.93 (0.73-1.17) | 0.92 (0.73-1.15) | 0.91 (0.73-1.15) |
| Beef | 147 | 159112 | 0.96 (0.76-1.22) | 0.97 (0.77-1.23) | 0.94 (0.74-1.20) |
| Visceral meat | 179 | 196857 | **1.25 (1.03-1.52)** | **1.24 (1.02-1.5)** | **1.24 (1.02-1.5)** |
| White meat | 161 | 209783 | 0.97 (0.79-1.21) | 0.96 (0.78-1.19) | 0.96 (0.78-1.19) |
| **CVD mortality** |  |  |  |  |  |
| Total meat | 30 | 157869 | 1.16 (0.7-1.92) | 1.24 (0.76-2.02) | 1.24 (0.75-2.04) |
| Total RM | 28 | 158143 | 1.07 (0.64-1.77) | 1.13 (0.69-1.83) | 1.13 (0.69-1.85) |
| Unprocessed RM | 30 | 158300 | 1.12 (0.69-1.84) | 1.18 (0.73-1.89) | 1.18 (0.72-1.91) |
| Processed RM | 20 | 138105 | 1.16 (0.72-1.87) | 1.19 (0.75-1.89) | 1.18 (0.74-1.87) |
| Pork | 28 | 158733 | 1.30 (0.79-2.14) | 1.41 (0.88-2.27) | 1.42 (0.88-2.3) |
| Pork belly | 41 | 170108 | **1.65 (1.07-2.55)** | **1.77 (1.16-2.69)** | **1.78 (1.17-2.71)** |
| Beef | 32 | 159112 | 1.07 (0.67-1.72) | 1.10 (0.70-1.74) | 1.09 (0.68-1.73) |
| Visceral meat | 47 | 196857 | 1.29 (0.90-1.86) | 1.31 (0.92-1.87) | 1.30 (0.91-1.86) |
| White meat | 39 | 209783 | 1.08 (0.71-1.63) | 1.07 (0.71-1.60) | 1.07 (0.71-1.6) |

a excluding the first 1 year of follow-up

b Adjusted for alcohol intake expressed in grams/day

c Further adjusted for intake of vegetables, fish, sea food and legumes

PY, person-years; RM, red meat
